# Neuromelanin-Sensitive MRI Contrast and Chronic Depression in Young Women

**DOI:** 10.1001/jamanetworkopen.2025.33339

**Published:** 2025-09-23

**Authors:** Greg Perlman, Roman Kotov, Kenneth Wengler, Scott J. Moeller, Guillermo Horga, Anissa Abi-Dargham, Daniel N. Klein

**Affiliations:** 1 Department of Psychiatry and Behavioral Health, Renaissance School of Medicine, Stony Brook University, Stony Brook, New York; 2 Department of Psychiatry, Icahn School of Medicine at Mount Sinai, New York, New York; 3 Department of Diagnostic, Molecular, and Interventional Radiology, Icahn School of Medicine at Mount Sinai, New York, New York; 4 Division of Translational Imaging, New York State Psychiatric Institute, New York, New York; 5 Department of Psychiatry, Columbia University, New York, New York; 6 Department of Psychology, Stony Brook University, Stony Brook, New York

## Abstract

**Importance:**

Whether chronic depression can be distinguished from nonchronic depression by mesolimbic dopamine hypofunction is a long-standing but untested hypothesis.

**Objective:**

To determine whether cumulative mesolimbic dopamine function, assessed with neuromelanin-sensitive magnetic resonance imaging (NM-MRI) contrast, is associated with chronic depression in young women.

**Design, Setting, and Participants:**

In the Adolescent Development of Emotions and Personality Traits (ADEPT) prospective cohort study, depressive disorders in women were assessed by diagnostic interview at regular intervals from ages 14 to 22 years (between July 2, 2012, and March 5, 2021). The current analysis includes a subset of ADEPT participants (aged 20-24 years) who completed a cross-sectional NM-MRI imaging study conducted between June 15, 2019, and May 31, 2021. Data were analyzed from March 27, 2024, to June 30, 2025.

**Main Outcomes and Measures:**

Course of depression was rated using semistructured interviews. The nonparametric permutation test (whole-mask voxelwise analysis) of NM-MRI contrast in 2060 voxels within the substantia nigra and ventral tegmental area complex was used to test the association of cumulative mesolimbic dopamine function with chronic depression.

**Results:**

Of the 166 young women contacted for this study, 140 had no MRI contraindications and were eligible. A total of 118 women underwent imaging, and 13 were excluded for MRI artifacts. Therefore, 105 women (mean [SD] age, 21.6 [0.91] years) were included in this analysis. There were 9 women (8.6%) with chronic depression (mean [SD], 45.6 [21.7] months), 28 (26.7%) with nonchronic depression (mean [SD], 6.0 [5.4] months), and 68 (64.8%) with no lifetime history of depression. Women with chronic depression exhibited significantly lower bilateral midbrain NM-MRI contrast than both women with nonchronic depression (795 negative-sign suprathreshold voxels, corrected *P* = .005) and women with no lifetime history of depression (692 negative-sign suprathreshold voxels, corrected *P* = .01), who did not differ from each other. Furthermore, lower bilateral midbrain NM-MRI contrast findings were associated with lower trait extraversion, a personality trait that reflects blunted reward sensitivity and is implicated in chronic depression, acquired at time of MRI (1061 positive-sign suprathreshold voxels, corrected *P* = .002), as well as from an earlier assessment at age 14 years prior to the onset of depression (1054 positive-sign suprathreshold voxels, corrected *P* = .001).

**Conclusions and Relevance:**

In this cohort study of young women, chronic depression was associated with reduced NM-MRI contrast, consistent with cumulative mesolimbic dopamine hypofunction. NM-MRI contrast findings were normal in young women with nonchronic depression. These findings support the importance of distinguishing between chronic and nonchronic forms of illness in parsing the heterogeneity of depressive disorders, and they suggest that low dopamine may play a role in the etiopathophysiology of chronic depression.

## Key Points

**Question:**

Does low dopaminergic function distinguish a chronic from nonchronic course of depression in young women aged 20 to 24 years?

**Findings:**

In this cohort study, 105 women for whom a course of depressive disorders was prospectively characterized from ages 14 to 22 years were recruited for assessment of neuromelanin-sensitive magnetic resonance imaging (NM-MRI), a marker of long-term midbrain dopamine function. Young women with a chronic course of depression were characterized by decreased NM-MRI contrast relative to those with nonchronic depression and those with no lifetime history of depression.

**Meaning:**

Results of this study suggest that low dopaminergic function plays a role in the etiopathophysiology of chronic depression.

## Teaser

This cohort study uses neuromelanin-sensitive magnetic resonance imaging contrast to examine the association between chronic depression and cumulative mesolimbic dopamine function in young women.

## Introduction

Depression is a heterogeneous phenotype, which presents a substantial challenge for efforts to derive targeted treatments from etiopathogenesis.^1^ One potential approach to reduce heterogeneity is to subtype by chronic and nonchronic courses of depression.^2^ Chronic depression is represented in current *Diagnostic and Statistical Manual of Mental Disorders* (*DSM*) nosology by persistent depressive disorder (formally labeled as dysthymia) and, in some cases, by the recurrent specifier for major depressive disorder (MDD), although the distinction is often difficult to diagnose reliably.^1^

There is increasing interest among translational scientists, clinicians, and diagnosticians to clarify the construct validity of chronic depression. Chronic depression differs from nonchronic depression on many clinical and psychosocial variables, including greater likelihood of experiencing early adversity and maltreatment, higher neuroticism and lower extraversion, and an elevated rate of depression in relatives.^2^ In 2023, several authors of the present study reported differences between women with chronic and nonchronic depression in premorbid personality, cognition, and interpersonal functioning.^3^ Importantly, there is also some evidence for qualitative differences. For example, chronic depression showed specific aggregation in families,^4,5,6^ and both chronic depression and episodic MDD remained stable, with little crossover, in long-term follow-up.^7^ In addition, Klein and Kotov^8^ showed a nonlinear increase in risk of poor long-term outcomes as a function of depression persistence, which indicates a distinct subgroup.

One notable gap in the emerging construct validation of chronic depression is evidence of distinctive etiopathogenesis. The Research Domain Criteria (RDoC) provide an integrative framework for organizing molecular, neural, and behavioral factors, referred to as biobehavioral dimensions. One RDoC dimension that may have utility for validating subtypes of depression is the positive valence system, which includes positive affect, reward processing, appetitive, and approach behavior. Extraversion, the behavioral-affective core of the positive valence system,^9^ is notably reduced in individuals with chronic depression relative to those with nonchronic MDD and individuals with no lifetime history of depression.^10,11^ Moreover, low extraversion has been reported in relatives of participants with chronic depression compared with relatives of those with nonchronic MDD,^12,13^ as well as in the premorbid state among those who later developed chronic depression.^3,14^ Finally, in several studies using event-related potentials, a blunted neural response to reward and positive stimuli was associated with a persistent course of depression in both individuals with depression^15^ and individuals with euthymia who later developed depression for the first time.^14^ Of note, dopaminergic neurotransmission is theorized to play a major role in the positive valence system, including extraversion.^9,16^ Thus, abnormalities in dopamine functioning may help distinguish chronic depression from nonchronic depression. This hypothesis is yet to be tested using standard molecular imaging techniques, such as positron emission tomography (PET), although one study of [^11^C]altropane reported a negative correlation between dopamine transporter binding and a retrospective estimate of number of lifetime depressive episodes.^17^ Instead, PET studies of dopaminergic function in depression have largely focused on case-control or pretreatment-posttreatment designs and yielded mixed or negative results overall.^18^

In this study, we examined mesostriatal dopamine function using an innovative neuroimaging modality: neuromelanin-sensitive magnetic resonance imaging (NM-MRI). NM-MRI quantifies NM concentration in brain tissue of living humans, which slowly accumulates across the lifespan via iron-dependent oxidation of cytosolic dopamine.^19^ Specifically, cytosolic dopamine that exceeds storage capacity in presynaptic vesicles can be oxidized, accumulate, and form NM in dopamine-producing neurons, such as in the substantia nigra (SN) and the ventral tegmental area (VTA).^20^ Importantly, NM in midbrain areas identifies the location of dopamine-producing neurons, which can be detected as NM-MRI contrast. Midbrain areas without NM accumulation are associated with low NM-MRI contrast (eg, where dopamine is not produced, such as GABAergic neurons). Furthermore, 2 cross-sectional PET studies in humans reported a direct association between increased NM-MRI contrast in the midbrain and increased dopamine function in vivo using [^11^C]-raclopride displacement in the striatum after amphetamine administration^21^ and dopamine synthesis capacity in the striatum and midbrain using [^18^F]-3,4-dihydroxyphenylalanine (DOPA).^22^ Although environmental factors associated with between- and within-individual differences in NM accumulation are not fully understood in humans, protracted use of addictive substances is associated with increased NM-MRI contrast.^23,24^ Studies in rodents suggest NM accumulation may be acutely accelerated by pharmacologic manipulation of dopamine biosynthesis (eg, administration of levodopa^25^). The inverse is also true; if cytosolic dopamine rarely exceeds storage capacity, there will be less conversion of cytosolic dopamine into NM in dopamine-producing neurons over time. Furthermore, NM accumulation in the midbrain is not cleared, except by the death of NM-containing neurons, such as in the case of neurogenerative diseases (eg, Parkinson disease). In this way, midbrain NM-MRI contrast results provide an easily acquired index of lifetime dopaminergic function that are suitable for testing the role of mesostriatal dopamine hypofunction in chronic depression.

The present sample was drawn from a cohort of women who were initially enrolled at age 14 years for the Adolescent Development of Emotions and Personality Traits (ADEPT) longitudinal prospective cohort study. First-onset depression and features of each episode (eg, duration) were assessed with repeated diagnostic interviews over the course of 8 years, allowing us to aggregate reports of depression into month-by-month life charts. In comparison, studies of chronic depression often rely on single whole-life retrospective assessment, which may raise concerns about the precision of such diagnoses.^26^ Approximately 8 years after baseline enrollment, we imaged women from this cohort with NM-MRI.

In this analysis, we explored 3 hypotheses. First, we hypothesized that participants with a history of chronic depression would exhibit reduced long-term dopaminergic function (eg, less NM-MRI contrast) than those with a history of nonchronic depression and a healthy comparison group. Second, given evidence of qualitative differences between chronic and nonchronic depression,^2,3^ we hypothesized that participants with nonchronic depression and no lifetime history of depression would not differ on NM contrast. Finally, because extraversion is thought to be the affective expression of a dopamine-mediated biobehavioral system,^9^ we hypothesized that extraversion would be positively associated with NM-MRI contrast.

## Methods

This cohort study was reviewed and approved by the institutional review board at Stony Brook University with written consent obtained from participants. This study was conducted in accordance with the Strengthening the Reporting of Observational Studies in Epidemiology (STROBE) reporting guideline for cross-sectional studies.

### Participants

Between June 15, 2019, and May 31, 2022, community-dwelling young women aged 20 to 24 years were recruited from the ongoing ADEPT study at Stony Brook University (550 women^14^) to participate in a cross-sectional NM-MRI study. eTable 1 in Supplement 1 presents sample bias analysis. Data were analyzed from March 27, 2024, to June 30, 2025. Women without known contraindications to MRI were screened by telephone for eligibility, and MRI acquisition was performed for those deemed eligible.^23^

Self-reported race and ethnicity were collected to assess demographic distributions using categories that would align with National Institutes of Health reporting mandates for race and ethnicity in human participant research.^27^ Race is reported as White or other race (including American Indian or Alaska Native, Asian, Black or African American, or >1 race), and ethnicity is reported as Hispanic or non-Hispanic. We report race as dichotomized because of low endorsement rates in some groups and to achieve better statistical power for detecting differences. Group differences were tested to examine demographic distributions. These categories were endorsed by participants.

### Measures

#### Depression

Depressive disorders were assessed by trained interviewers (between July 2, 2012, and March 5, 2021) using a modified version of the *DSM-IV* Kiddie Schedule for Affective Disorders and Schizophrenia for School-Age Children–Present and Lifetime Versions.^28^ Our primary hypotheses focused on a 3-group categorical variable (chronic depression, nonchronic depression, or no lifetime history of depression) based on a cutoff of 24 months of depression in a lifetime (eg, meeting criteria for MDD, dysthymia, or both). Complementary analyses using a dimensional variable (depression months) based on total months of meeting criteria for MDD, dysthymia, or both in a lifetime are also presented. Further details are presented in the eMethods in Supplement 1.

#### Extraversion

We used the 6-item extraversion scale from the Big Five Inventory,^29^ a 44-item, factor-analytically derived measure of personality. Each item consists of short descriptive phrases that are rated on a 5-point Likert scale ranging from 1 (disagree strongly) to 5 (agree strongly). Additional details are presented in the eMethods in Supplement 1.

### Imaging and NM-MRI Data Processing

We followed previous work^30^ with a validated pipeline^21^ to preprocess NM-MRI image data and calculate the NM contrast ratio. Quality control procedures were followed; individuals were excluded from this study if MRI artifacts were present. Complete data processing details are presented in the eMethods in Supplement 1.

### Statistical Analysis

Whole-mask voxelwise analysis with nonparametric permutation testing was used to test the primary hypothesis of a between-group difference in NM-MRI contrast for the 3-group chronic depression variable as well as depression months. In brief, a count of voxels that surpassed a given height threshold was calculated for the variable of interest within an a priori SN-VTA mask (eg, 1-sided *P* < .05 in the voxelwise analysis).^21^ The null distribution was created from supraheight threshold voxel counts from 10 000 voxelwise analyses using random shuffles of the variable of interest. A corrected *P* value is thus the percentile rank of the real data supraheight threshold voxel count relative to the null distribution. For associations, we also report the leave-one-out Pearson correlation (*r*_LOO_), an unbiased measure of effect size.^21,31^

Complementary analyses using information from all voxels in the SN-VTA mask were designed to better describe NM-MRI contrast in relation to chronic depression. First, we repeated the main analysis using a region-of-interest (ROI) approach to test whether the mean NM-MRI contrast of all 2060 voxels in the SN-VTA mask was associated with the 3-group chronic depression variable as well as for the dimensional variable, respectively. We present the ROI analyses for the whole mask as well as for the following midbrain subnuclei whose locations were previously estimated by a probabilistic atlas at a threshold of 50%^32^: VTA, SN pars compacta (SNc), SN pars reticulata (SNr), parabrachial pigmented nucleus (PBP), and other SN (all remaining voxels in the mask). Second, we examined the association between voxel-level NM-MRI contrast from the grand mean and the voxel-level inferential statistic with depression months (*t* statistic) from the voxelwise analysis. For instance, a positive association indicates that chronic depression is strongly associated with NM-MRI contrast in midbrain voxels that have high contrast (eg, indicative of more NM accumulation). Third, we plotted the distribution of suprathreshold voxels by the a priori–defined subnuclei masks. The distribution of suprathreshold voxels may help identify specific dopamine pathways underlying abnormal levels of NM accumulation in chronic depression (eg, mesolimbic pathway or nigrostriatal pathway).

Of note, several authors of the present study previously reported a positive association between cumulative substance use history and midbrain NM-MRI contrast in the present sample.^23^ Therefore, we conducted supplemental analyses while controlling for cumulative substance use (eMethods and eTable 5 in Supplement 1).

Data analysis was performed with R, version 4.4.1 (R Project for Statistical Computing), and Matlab, version 2022a (MathWorks). MRIcroGL, version 1.2.20220720, was used to generate magnetic resonance images.^33^

## Results

### Sample Characteristics

**Figure 1.**
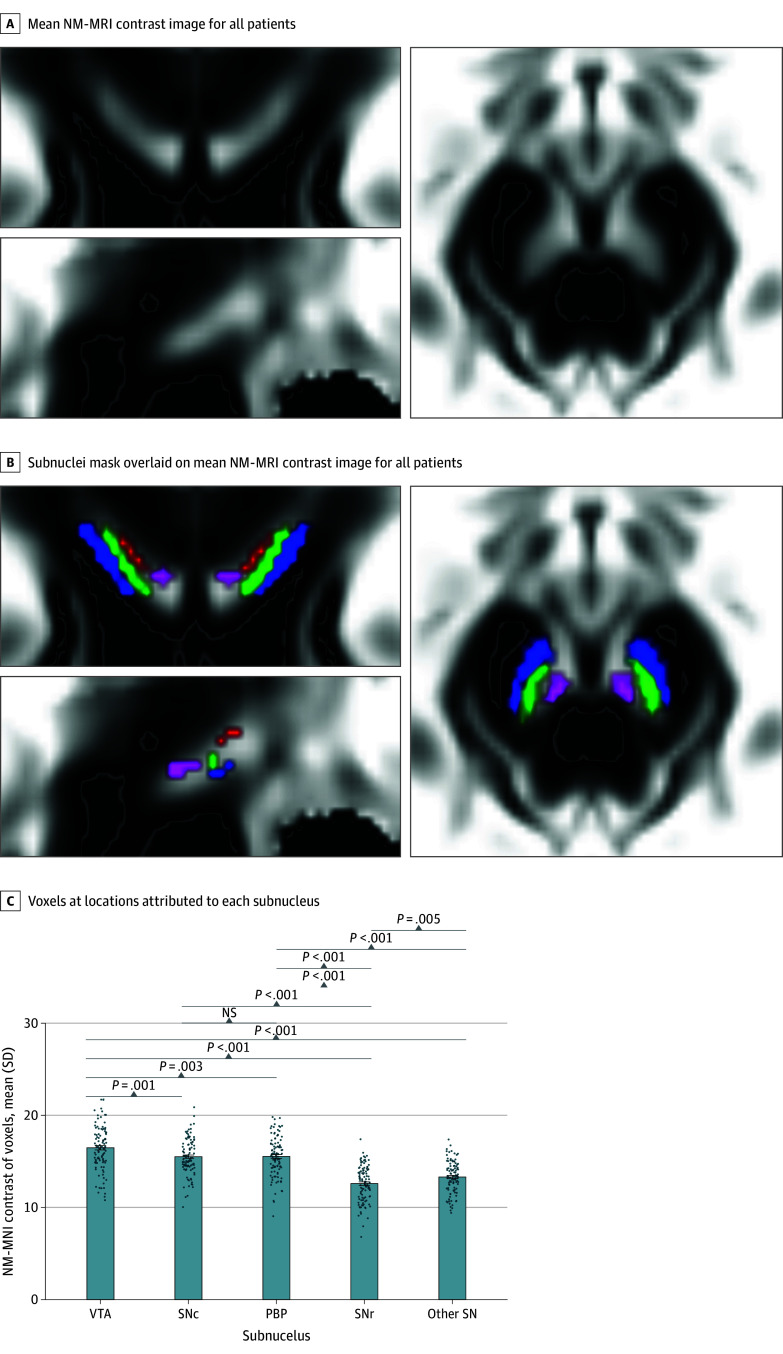
Neuromelanin-Sensitive Magnetic Resonance Imaging (NM-MRI) Contrast in Women Aged 20 to 24 Years A, The grand mean NM-MRI contrast image from all participants at coronal (top left), axial (bottom), and sagittal (top right) views at Montreal Neurological Institute (MNI) coordinates 84, 106, 56; 95, 106, 56; and 83, 110, 56. B, Grand mean NM-MRI contrast image as in A with the subnuclei mask overlaid from coronal (top left), axial (bottom), and sagittal (top right) views at MNI coordinates 84, 106, 56; 95, 106, 56; and 83, 110, 56, respectively. Blue indicates substantive nigra pars reticulata (SNr); green, substantia nigra pars compacta (SNc); red, parabrachial pigmented nucleus (PBP); and purple, ventral tegmental area (VTA). C, Mean NM-MRI contrast of voxels at locations attributed to each subnucleus. Error bars were calculated as 1 times the SE. Other SN includes remaining voxels in the SN-VTA mask that had low probability of inclusion in the other 4 subnuclei. Dots are the mean NM-MRI contrast for VTA, SNr, SNc, PBP, and other SN ROIs for each participant. NS indicates not significant.

A total of 166 young women in the ADEPT study without known contraindications to MRI were screened by telephone to determine eligibility for this study. Of the 140 women deemed eligible, 118 successfully completed MRI acquisition.^23^ Quality control procedures excluded 13 women for MRI artifacts, resulting in a final sample of 105 participants (mean [SD] age, 21.6 [0.91] years). Participants identified as White (98 [93.3%]) or other race (7 [6.7%]) and as Hispanic (9 [8.6%]) or non-Hispanic (96 [91.4%]). The Table summarizes the sample characteristics by group, including demographics, depression course, and trait extraversion. Applying the categorical definition, there were 9 participants (8.6%) with chronic depression, 28 (26.7%) with nonchronic depression, and 68 (64.7%) with no lifetime history of depression. The mean (SD) duration of depression was 45.6 (21.7) months for the chronic depression group and 6.0 (5.4) months for the nonchronic depression group, respectively. Figure 1 depicts the grand mean NM-MRI contrast (105 women) in Montreal Neurological Institute (MNI) space as well as location and contrast by subnuclei (eFigure 1 in Supplement 1).

**Table.** Participant Demographic Characteristics^a^

Characteristic	Participants (N = 105)			χ^2^ or *F* value	*P* value
	No lifetime history of depression (n = 68)	Nonchronic depression (n = 28)	Chronic depression (n = 9)		
Race					
White	62 (91.2)	27 (96.4)	9 (100)	1.58^b^	.45
Other race^c^	6 (8.8)	1 (3.6)	0		
Ethnicity					
Hispanic	6 (8.8)	2 (7.1)	1 (11.1)	0.15^b^	.93
Non-Hispanic	62 (91.2)	26 (92.9)	8 (88.9)		
Psychiatric medication use					
Any lifetime	9 (15.5)	9 (28.6)	8 (55.6)	25.53^b^	<.01
SSRIs	3 (4.4)	8 (32.1)	6 (75.0)		
NDRIs	1 (1.5)	0	2 (22.2)		
Tricyclic antidepressants	0	1 (3.6)	0		
Atypical antipsychotic	0	1 (3.6)	0		
Stimulants	3 (4.4)	1 (3.6)	1 (11.1)		
Barbiturates or benzodiazepines	4 (5.9)	0	2 (22.2)		
Opioids	2 (2.9)	0	1 (11.1)		
Any pharmaceutical contraceptive use	32 (47.1)	12 (42.9)	4 (44.4)	0.14^b^	.93
Age, mean (SD), y					
At wave 1	14.4 (0.7)	14.2 (0.6)	14.2 (0.6)	1.07^d^	.35
At MRI acquisition	21.7 (1.0)	21.4 (0.7)	21.5 (1.0)	1.25^d^	.29
Depression months	0	6.0 (5.4)	45.6 (21.7)	NA	NA
Extraversion, mean (SD)					
At wave 1	23.1 (5.5)	22.8 (3.7)	19.8 (5.8)	1.69^d^	.19
At MRI	21.9 (5.2)	21.9 (4.5)	15.4 (5.4)	6.72^d^	<.01
Household income at wave 1, mean (SD)^e^	6.7 (1.7)	6.4 (1.5)	6.1 (1.5)	0.79^d^	.46
Cumulative substance use, mean (SD)^f^	−0.2 (0.9)	0.4 (1.0)	−0.3 (1.0)	4.20^d^	.02
Physical health, mean (SD)^g^	4.2 (0.7)	3.7 (0.7)	3.4 (1.3)	7.60^h^	<.01
NM-MRI quality, mean (SD)^i^					
Approved volumes	10.3 (1.6)	10.1 (2.0)	10.2 (1.3)	0.33^d^	.72
Framewise displacement	1.6 (1.0)	1.6 (0.8)	1.9 (0.6)	0.19^d^	.83

Abbreviations: MRI, magnetic resonance imaging; NA, not applicable; NDRI, norepinephrine-dopamine reuptake inhibitor; NM-MRI, neuromelanin-sensitive magnetic resonance imaging; SSRI, selective serotonin reuptake inhibitor.

^a^ Unless indicated otherwise, values are presented as No. (%) of participants.

^b^ From χ^2^_2_ value.

^c^ Includes American Indian or Alaska Native, Asian, Black or African American, or more than 1 race.

^d^ *F*_2102_ value.

^e^ Household income was rated by the participating parent at baseline (wave 1) on a 9-point ordinal scale ranging from 1 (<$20 000) to 9 (>$180 000).

^f^ Medication status was compiled from semistructured interview data at wave 6 and at MRI. See the eMethods in Supplement 1 for a description of cumulative substance use.

^g^ Physical health was rated on a 5-point scale ranging from 1 (very bad) to 5 (very good). One participant in the group with no lifetime history of depression did not complete the physical health assessments.

^h^ *F*_2101_ value.

^i^ The maximum number of approved volumes was 11. Framewise displacement refers to the average displacement for the approved volumes as estimated during realignment.

### Categorical Analysis of the Association Between NM-MRI Contrast and Chronic Depression Groups

**Figure 2.**
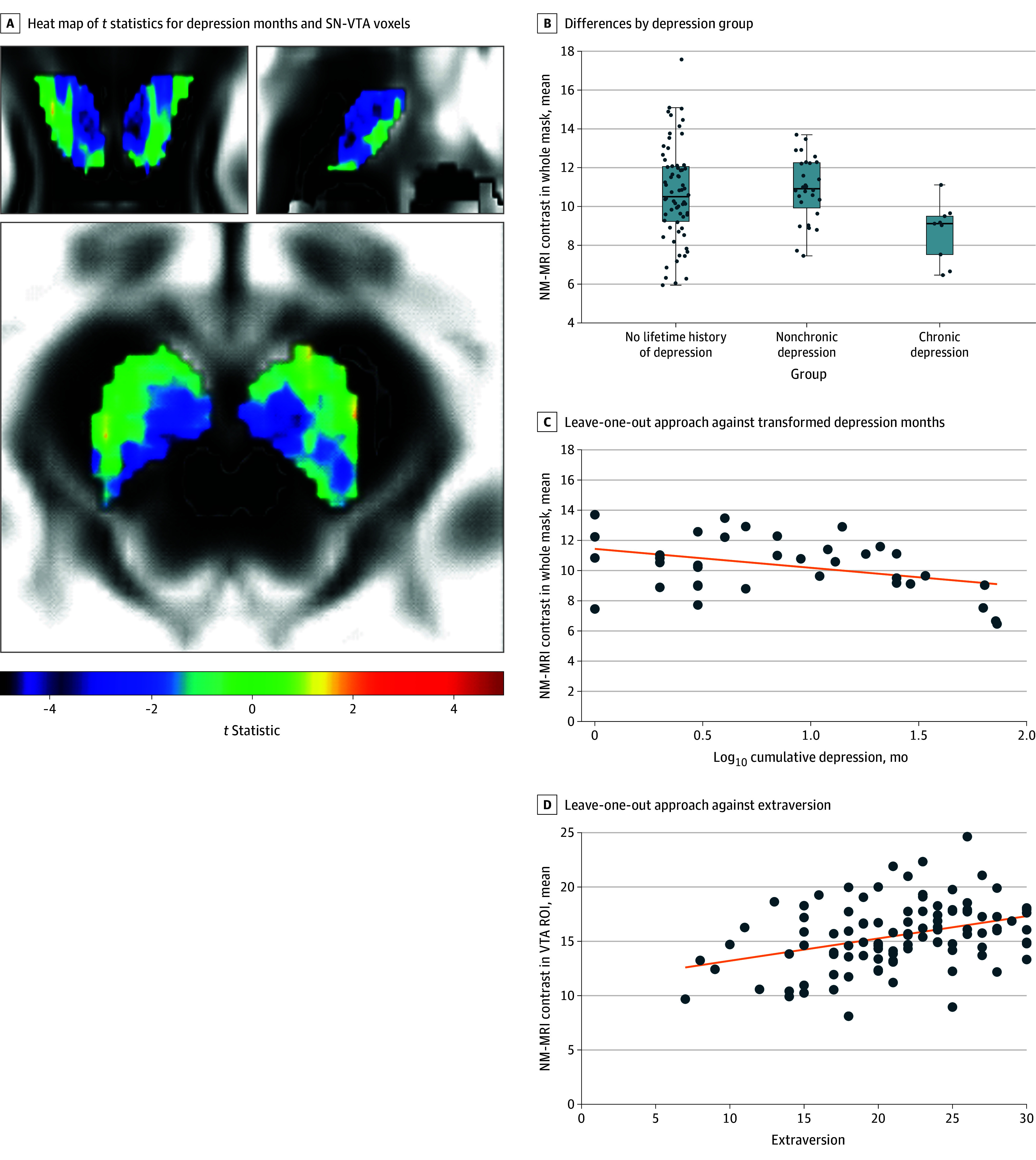
Neuromelanin-Sensitive Magnetic Resonance Imaging (NM-MRI) Contrast and Chronicity of Depression A, Heat map of *t* statistics depicting the association between log_10_-transformed depression months and voxels in the substantia nigra (SN) and the ventral tegmental area (VTA) controlling for age overlaid on the average NM-MRI contrast image from all participants at Montreal Neurological Institute (MNI) coordinates 87, 108, 56 from axial (left side), coronal (middle), and sagittal (right side) views. B, Group differences in the mean NM-MRI contrast in SN-VTA mask (region of interest [ROI]) by depression group (eTable 2 in Supplement 1). Each dot is one participant’s mean NM-MRI contrast. The box is drawn around the median of the lower half quartile and the median of the upper half quartile. The error bars are drawn to the first and last value, excluding outliers. C and D, Scatterplot showing the mean NM-MRI contrast extracted from suprathreshold voxels in Figure 1A using a leave-one-out approach plotted against log_10_-transformed depression months (C) and extraversion (D) for visualization purposes. Each dot is one participant's data. The line is the ordinary lease squares regression. It demonstrates the slope of the association between the two variables

In the 3-group analysis of covariance model, there was an association between depression group and NM-MRI contrast (450 supraheight threshold voxels, corrected *P* = .02). Per pairwise contrasts, the chronic depression group had significantly lower NM-MRI contrast compared with the group with no lifetime history of depression (1 positive-sign suprathreshold voxel, corrected *P* = .98; and 692 negative-sign suprathreshold voxels, corrected *P* = .01; *r*_LOO_ = −0.27) and nonchronic depression group (0 positive-sign suprathreshold voxels and 795 negative-sign suprathreshold voxels, corrected *P* = .005; *r*_LOO_ = −0.48). The latter 2 groups did not differ from each other (44 positive-sign suprathreshold voxels, corrected *P* = .51; and 32 negative-sign suprathreshold voxels, corrected *P* = .60). The same pattern was observed in ROI analysis (Figure 2A and eTable 2 in Supplement 1).

### Dimensional Analysis

#### Association Between NM-MRI Contrast and Depression Months

Consistent with the group analyses, depression months (log_10_ transformed due to skew) was negatively correlated with NM-MRI contrast in the voxelwise analysis. The higher the depression chronicity, the lower the NM-MRI contrast (2 positive-sign suprathreshold voxels, corrected *P* = .98; and 756 negative-sign suprathreshold voxels, corrected *P* = .01; *r*_LOO_ = −0.44) (Figure 2B and C and eFigure 2 in Supplement 1). The same pattern was observed in ROI analysis (eTable 3 in Supplement 1). As illustrated in eFigure 3 in Supplement 1, suprathreshold voxels (756 of 2060 [36.7%]) exhibited greater NM-MRI contrast relative to subthreshold voxels (1304 of 2060 [63.3%]), indicating stronger associations in voxels with greater contrast.

#### Association Between NM-MRI Contrast and Extraversion

In the voxelwise analysis, NM-MRI contrast was positively associated with trait extraversion acquired at the time of the MRI scan (concurrent association) (1061 positive-sign suprathreshold voxels, corrected *P* = .002, *r*_LOO_ = 0.29; and 8 negative-sign suprathreshold voxels, corrected *P* = .84) (Figure 2D). This association was also observed for trait extraversion prior to the onset of depression at wave 1 (concurrent association) (1054 positive-sign suprathreshold voxels, corrected *P* = .001, *r*_LOO_ = 0.29; and 4 negative-sign suprathreshold voxels, corrected *P* = .99). The same pattern was observed in ROI analysis (eTable 4 in Supplement 1).

### Distribution of Significant Voxels by Subnuclei Mask

**Figure 3.**
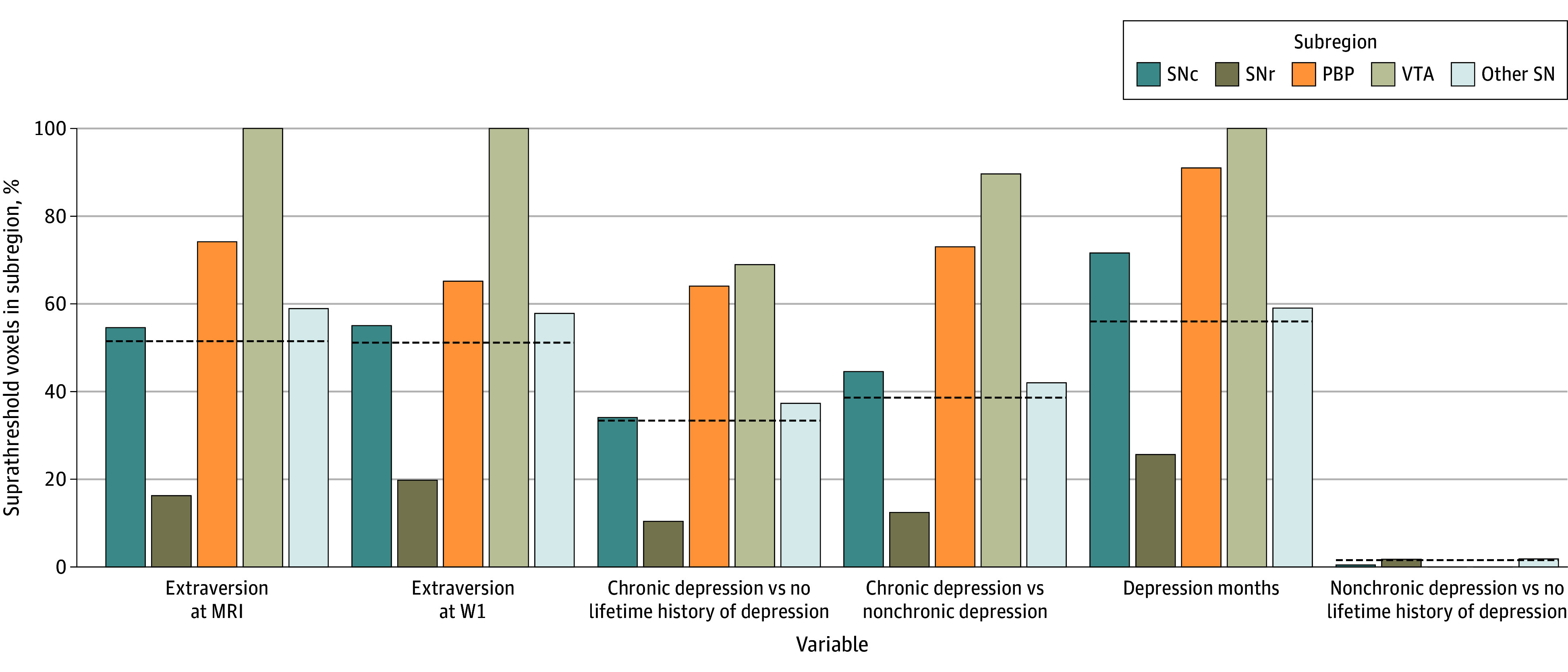
Proportion of Significant Voxels in Subregion Reference dashed lines are drawn at the proportion of supraheight threshold voxels in whole-mask voxelwise analysis (2060 voxels). Bars that surpass the dashed lines indicate that more voxels in that subnuclei mask were supraheight threshold than expected by chance, given the total number of supraheight threshold in mask. Bars below the dashed lines indicate that fewer voxels in that subnuclei mask were supraheight threshold than expected by chance, given total number of supraheight threshold in mask. For instance, the reference line is drawn at 0.37 (756 of 2060) for log_10_-transformed depression months, which is the proportion of negative-sign supraheight threshold voxels from voxelwise analysis. The dashed line corresponds to the positive-sign supraheight threshold voxels for extraversion variables, the negative-sign supraheight threshold voxels for depression months, and the supraheight threshold voxels for group comparisons (no lifetime history of depression > chronic depression, no lifetime history of depression > nonchronic depression, and nonchronic depression > chronic depression). Voxels at the ventral tegmental area (VTA) coordinates, as well as the substantia nigra pars compacta (SNc) and parabrachial pigmented nucleus (PBP) coordinates, were disproportionately supraheight threshold for chronic depression and extraversion. Voxels in the substantive nigra pars reticulata (SNr) subnuclei coordinates were not disproportionately supraheight threshold for chronic depression and extraversion. Other SN indicates remaining voxels in the SN-VTA mask that had low probability of inclusion in the other 4 subnuclei. MRI indicates magnetic resonance imaging; W1, wave 1.

Figure 3 illustrates the proportion of suprathreshold voxels by subregion for depression variables and extraversion variables from their respective voxelwise analysis. As shown, there was a relatively similar spatial pattern of suprathreshold voxels by subnuclei. This pattern included more than expected suprathreshold voxels in locations identified as the VTA and PBP and fewer than expected suprathreshold voxels in the SNr. Complementary ROI analyses and sensitivity analyses are presented in the eResults in Supplement 1.

## Discussion

The role of midbrain dopamine function in depression in general, and in chronic depression in particular, is poorly understood.^17,18,34,35^ Yet it is critically important to understand, given the magnitude of chronic depression’s public health burden and the need for novel therapeutic strategies. To address this gap in knowledge, we acquired an index of lifetime midbrain dopamine function in 105 young women (aged 20-24 years) from the community whose depression history was traced prospectively over 8 years.

Our first main finding is that NM-MRI contrast was significantly lower among young adult women with chronic depression than among both their peers with nonchronic depression and no lifetime history of depression, who did not differ from each other. This result was robust to 2 analytic approaches (voxelwise and ROI) and to 2 definitions of chronic depression (categorical and dimensional), and it remained significant when controlling for lifetime substance use (eTable 5 in Supplement 1). The second main finding is that midbrain NM-MRI contrast was positively correlated with trait extraversion. Extraversion, the affective expression of the positive valence system,^36,37^ is thought to be influenced by dopamine-rich neural circuits^9^ and has been linked to chronic depression in past studies.^3,10,11,12,14,15^ However, these data are the first, to our knowledge, to show an association between low trait extraversion and low dopamine function indexed by NM-MRI contrast. This study is also the first, to our knowledge, to suggest that chronic depression and low extraversion share similar midbrain dopamine pathways, as corroborated by similarity in the pattern of results by subnuclei. These findings support the notion that chronic depression is associated with dysregulation of the positive valence system, whereas there was no evidence of this association in nonchronic depression.

The link between chronic depression and decreased dopamine function has important implications for translational and etiological models. For instance, the data from the current study suggest that chronic depression is linked to hypodopaminergic function in pathways characterized by greater dopaminergic biosynthesis. This includes the VTA, the primary source of dopaminergic projections to the ventral striatum (including the nucleus accumbens), which forms a pathway involved in processing reward cues and salience, as well as to the prefrontal cortex, which helps to govern cognitive functions such as working memory.^38^ The PBP is also a dopamine-rich area that projects to the ventral striatum.^39,40^ One consequence of reduced midbrain dopamine biosynthesis is to disrupt dopamine signaling in the striatum and thus affect dopaminergic-mediated processes, such as reward sensitivity and reinforcement learning.^41,42^ Over time, anhedonic symptoms (eg, diminished motivation or social withdrawal) may emerge and contribute to a depressogenic positive feedback loop that acts to maintain depressive symptoms.^43^ Importantly, NM-MRI techniques thus appear suitable for indexing cumulative dopamine biosynthesis within the mesolimbic and corticolimbic pathways.

The results of this study also have implications for public health. To the extent that low extraversion during adolescence is an index of low dopamine function, then it may prove cost-effective to use extraversion in screening efforts for high-risk youths for dopaminergic-enhancing treatments. Similarly, targeted treatments for ameliorating low dopaminergic function early in the course of depression may help increase rates of remission in adolescent-onset depression. Of note, baseline extraversion was collected prior to the onset of depression in this sample, ruling out scar effects of depression that may bias self-report personality ratings.^44^ However, it is not yet clear whether decreased NM-MRI contrast distinguishes youths who will later develop chronic depression (eg, predicts duration of depressive episodes) or reflects progressive deterioration of dopamine function related to protracted depressive episodes. To our knowledge, no prior molecular imaging studies of dopamine function in depression have examined chronicity of depression per se, although Pizzagalli et al^17^ reported that more episodes of depression in adults were associated with decreased dopamine signaling. Although molecular imaging could offer key insights into changes in dopamine function in vivo before onset and during chronic depression, this imaging method may not be used in research with high-risk children and adolescents. Therefore, the development and validation of dopamine imaging strategies, such as NM-MRI, that are noninvasive and capable of describing dopamine-disease associations that begin in childhood and unfold over time are critically important. Future longitudinal studies with repeated NM-MRI assessments, especially in younger cohorts, are necessary to test whether decreased dopamine biosynthesis predates, follows, or is modulated by chronic depression.

### Limitations

There are limitations to the current study. First, it will be important to replicate these findings in larger samples of individuals with chronic depression, especially in men. Second, the cross-sectional imaging design obscures the temporal order of association between NM-MRI contrast, depression onset, and extraversion. For instance, the long-term consequence of midbrain hypodopaminergic function on development of the positive valence system is unclear. It is also unclear whether common medications used to treat depression affect the rate of NM accumulation (eg, iatrogenic effects). Developmentally informative imaging studies are needed to test the temporal associations between depression, extraversion, and NM-MRI contrast. Third, the study design could not distinguish among competing mechanisms for decreased NM accumulation in chronic depression, such as decreased dopamine synthesis or disrupted dopamine storage capacity by vesicular monoamine transporters. Fourth, we used a previously validated approach to operationalize chronicity of depression (cumulative time depressed since onset).^45,46^ Although these findings add to the growing literature that supports chronic depression as a clinically important construct, its operationalization remains open. Finally, although community sampling was a strength of our study, the results may not generalize to key populations of interest, such as treatment-seeking youths, including those with comorbid psychiatric or medical conditions.

## Conclusions

In this cohort study of young women, NM-MRI showed that chronic depression is associated with midbrain hypodopamine function. These data generally support the importance of distinguishing between chronic and episodic forms of illness in parsing the heterogeneity of depressive disorders for enhancing precision medicine.

## Notes

Supplement 1.
eMethods

eResults

eReferences
**eTable 1.** Sample Bias Analysis **eTable 2.** Region-of-Interest Analysis for Depression Groups **eTable 3.** Region-of-Interest Analysis for Depression Months **eTable 4.** Region-of-Interest Analysis for Extraversion **eTable 5.** Group Differences in Substance Use **eFigure 1.** Mosaic Displays of Grand Average Neuromelanin-Sensitive Magnetic Resonance Imaging (NM-MRI) Contrast From Axial, Coronal, and Sagittal Views **eFigure 2.** Mosaic Displays of Neuromelanin-Sensitive Magnetic Resonance Imaging (NM-MRI) Contrast From Axial, Coronal, and Sagittal Views With a *t*-Statistic Map From Voxelwise Analysis **eFigure 3.** Histogram of Significant Voxels From Association Between Neuromelanin-Sensitive Magnetic Resonance Imaging (NM-MRI) Contrast and Depression Months

Supplement 2.
Data Sharing Statement
