# Supplementary material for: Neuromelanin-Sensitive MRI Contrast and Chronic Depression in Young Women
Source: JAMA Netw Open. 2025 Sep 23;8(9):e2533339. doi: 10.1001/jamanetworkopen.2025.33339 (PMC12457983; doi:10.1001/jamanetworkopen.2025.33339)
Supplement: Supplement 1. — eMethods eResults eReferences eTable 1. Sample Bias Analysis eTable 2. Region-of-Interest Analysis for Depression Groups eTable 3. Region-of-Interest Analysis for Depression Months eTable 4. Region-of-Interest Analysis for Extraversion eTable 5. Group Differences in Substance Use eFigure 1. Mosaic Displays of Grand Average Neuromelanin-Sensitive Magnetic Resonance Imaging (NM-MRI) Contrast From Axial, Coronal, and Sagittal Views eFigure 2. Mosaic Displays of Neuromelanin-Sensitive Magnetic Resonance Imaging (NM-MRI) Contrast From Axial, Coronal, and Sagittal Views With a t-Statistic Map From Voxelwise Analysis eFigure 3. Histogram of Significant Voxels From Association Between Neuromelanin-Sensitive Magnetic Resonance Imaging (NM-MRI) Contrast and Depression Months [file jamanetwopen-e2533339-s001.pdf]

Supplemental Online Content

Perlman G, Kotov R, Wengler K, et al. Neuromelanin-sensitive MRI contrast and chronic depression in young women. *JAMA Netw Open*. 2025;8(9):e2533339. doi:10.1001/jamanetworkopen.2025.33339

eMethods

eResults

eReferences

eTable 1. Sample Bias Analysis

eTable 2. Region-of-Interest Analysis for Depression Groups

eTable 3. Region-of-Interest Analysis for Depression Months

eTable 4. Region-of-Interest Analysis for Extraversion

eTable 5. Group Differences in Substance Use

eFigure 1. Mosaic Displays of Grand Average Neuromelanin-Sensitive Magnetic Resonance Imaging (NM-MRI) Contrast From Axial, Coronal, and Sagittal Views

eFigure 2. Mosaic Displays of Neuromelanin-Sensitive Magnetic Resonance Imaging (NM-MRI) Contrast From Axial, Coronal, and Sagittal Views With a *t*-Statistic Map From Voxelwise Analysis

eFigure 3. Histogram of Significant Voxels From Association Between Neuromelanin-Sensitive Magnetic Resonance Imaging (NM-MRI) Contrast and Depression Months

This supplemental material has been provided by the authors to give readers additional information about their work.

© 2025 Perlman G et al. *JAMA Network Open*.

eMethods

Sample

Inclusion criteria for the Adolescent Development of Emotions and Personality Traits (ADEPT) study at Stony Brook University at baseline (or “Wave 1”) were age 13.5-15.5, English fluency, ability to complete questionnaires, and a biological parent willing to participate. Exclusion criteria at baseline were intellectual disability and lifetime history of MDD or dysthymia at study entry. All participants provided written informed consent after the procedures had been fully explained.

© 2025 Perlman G et al. *JAMA Network Open*.

Analysis of Sample Bias

Analysis of attrition bias between 118 women that completed an NM-MRI scan and remaining 432 women from the ongoing longitudinal study revealed minimal group differences on key study variables, such as diagnosis of chronic depression or trait extraversion. However, the MRI sample was somewhat more likely to identify as Caucasian (94% vs 86%), and their parents reported a higher family income at Wave 1 (eTable 1).

© 2025 Perlman G et al. *JAMA Network Open*.

Measurement of Chronic and non-Chronic Depression

At baseline, disorders were assessed for the participant's lifetime. At each subsequent follow-up (Waves 2-5 were completed at approximately 9 month intervals; Wave 6 was completed approximately 36 months after Wave 5), disorders were assessed since the last assessment. No participants with NM-MRI data were excluded for missing Depression Diagnosis data. Kappas for MDD and dysthymic disorder across raters were 0.73 and 0.85, respectively. Following recommendations[1], we used life chart methods to construct month-by-month ratings of depression throughout the follow-up period. Chronicity of depression was thus derived using two indices – one categorical and one dimensional.

The *categorical definition of chronic depression* was meeting DSM-IV criteria for MDD and/or dysthymia for a cumulative total of at least 24 months during the 8-year follow-up period. This differed from the standard DSM-IV criteria for dysthymia (and DSM-5 criteria for persistent depressive disorder) in that it allowed for frequent depressive episodes totaling at least 24 months. As discussed elsewhere[2,3], several studies have demonstrated, using familial aggregation of depression as an external criterion, that cumulative duration of depression since onset provides a more valid and parsimonious definition of chronic depression than DSM critiera.[4,5] Non-chronic depression was defined as meeting criteria for MDD and/or dysthymia for a cumulative duration of less than 24 months. Lastly, the never depressed group was defined as never meeting criteria for MDD and/or dysthymia in lifetime. The groups were dummy coded as no history of depressive disorder ("0"), non-chronic depression ("1"), and chronic depression (coded "2"), respectively.

The *dimensional definition of chronic depression* was defined as the total number of months in which the participant met criteria for MDD and/or dysthymia. For clarity, we use the term "Depression Months" throughout the manuscript when referring to this variable. The non-depressed group was excluded from dimensional analyses. Due to a few outliers on Depression Months (e.g., especially large number of months with MDD and/or dysthymia), we present

results for log10-transformed Depression Months in the main text and untransformed Depression Months in Online Material. The substantive interpretation of results was unchanged by transformation.

© 2025 Perlman G et al. *JAMA Network Open*.

Assessment of Extraversion

BFI Extraversion was assessed at same visit as depression diagnosis: Waves 2-5 were completed at approximately 9 month intervals after Wave 1; Wave 6 was completed approximately 36 months after Wave 5. Extraversion was also completed at the MRI visit after Wave 6. The BFI has demonstrated good internal consistency, test-retest reliability, and convergent and discriminant validity[6,7]. The present study focused on the extraversion scale (6 items), which was available at the study baseline (α = .81) and at the time of MRI (α = .87). The test-retest correlation between Extraversion measured at baseline and extraversion measured at the time of MRI was $r = 0.68$, $n=105$, $p <0.001$. No participants with NM-MRI data were excluded for missing Extraversion data. The other four scales of the BFI (neuroticism, agreeableness, conscientiousness, and openness to experience) were assessed at all waves, but not considered for inclusion in the present study.

© 2025 Perlman G et al. *JAMA Network Open*.

Image Acquisition

Participants were screened over the phone for MRI-contraindications (e.g., metal implants, claustrophobia, history of head injuries, etc.) and, if eligible, were scheduled for an NM-MRI acquisition. Image acquisition was completed in approximately 60 minutes using a Siemens PRISMA 3T scanner with Siemens 64-channel head/neck coil. T1w structural images were acquired using a 3D-MPRAGE sequence with the following parameters: TR/TE/TI =2400/2.24/1060 ms, FOV=256, voxel size=0.8x.0.8x0.8 mm3, flip angle=8°, slices=208, and GRAPPA parallel imaging factor=2. The NM-MRI sequence (11 sequential volumes) consisted of 2D gradient echo (GRE) images with magnetization transfer contrast of the midbrain (2D-GRE-MT, 0.39 mm2 in-plane resolution, 10 slices, FOV=162×200 mm, FA=40°, TR= 260 ms, TE=2.68 ms, MT frequency offset=1200 Hz, AC-PC alignment). NM-MRI slice prescription followed published recommendations.[8]

© 2025 Perlman G et al. *JAMA Network Open*.

NM-MRI data processing.

For each participant, we followed published guidelines for excluding motion artifacts and other artifacts affecting the midbrain[8]. All 11 volumes were approved for 82 participants and between 5 and 10 high quality volumes were approved for 23 participants. Thirteen women were excluded for having 4 or fewer high quality volumes[9]. Hence, our analysis sample was n = 105. The T1w image was warped into MNI space using Advanced Normalization Tools (ANTs). Each NM-MRI volume was realigned to the first volume using ANTs and then averaged. The average NM-MRI image was then warped into MNI space by co-registration to the warped T1w image and smoothed using a 1mm FWHM Gaussian smoothing kernel. For each subject, each voxel at location (x,y,z) was normalized within-subject by conversion to a contrast ratio[9], defined as: $voxel(x,y,z) = 100 * [I(x,y,z) – mode_{ref}] / mode_{ref}$, where I(x,y.z) denotes the signal intensity of a voxel located at position (x,y,z) and $mode_{ref}$ denotes the mode signal within a neighboring structure known from anatomical studies to contain no neuromelanin (i.e., the Crus Cerebri). The SN-VTA mask (2060 voxels) and Crus Cerebri mask (761 voxels) were hand-drawn and spatially-normalized in MNI space[9,10]. Prior to voxelwise analysis, contrast outlier values that were greater than the top 1% or less than the bottom 1% of the distribution were excluded from analysis to enhance rigor and minimize effects of rare outlier values.[10] Of the 216,300 in-mask voxels that could be included in analyses (105 subjects x 2060 voxels), the criteria resulted in exclusion of 4,326 extreme voxels (1% less than -5.4505 contrast and 1% greater than 27.3412 contrast). This left 211,974 voxels for analysis across 105 participants.

© 2025 Perlman G et al. *JAMA Network Open*.

NM-MRI analyses

For whole-mask voxelwise analysis of NM-MRI contrast, the spatial extent of an effect was defined as the number of supra-height threshold voxels *k* (adjacent or nonadjacent) for a variable of interest across all voxels in the SN-VTA mask (n=2060). For ANOVA/ANCOVA models, k is calculated as the count of voxels for which the F-statistic (main effect) for the variable of interest (e.g., Depression Groups) surpassed a voxel-level height threshold of $p < 0.05$. For dimensional analyses, such as for Depression Months (substituted in lieu of the three-level categorical Depression Group variable), Extraversion, and post-hoc pairwise group comparisons (e.g., Chronic Depression vs Non-Chronic Depression), we used linear regression models. In such models, k is calculated as the count of voxels for which the test-statistic (e.g., beta, t-test, etc.) surpassed a voxel-level height threshold of $p < 0.05$, one-sided, separately for positive-sign voxels and negative-sign voxels.

For all voxelwise analyses (ANCOVA and linear regression), hypothesis testing was performed by computing the probability ($p_{corrected}$) of observing *k* (the real data supra-threshold voxel count) relative to a null distribution of “randomized data” supra-threshold voxel counts created by permutation test. Importantly, the non-parametric permutation test involved shuffling the variable of interest across individuals and re-running the voxelwise analysis 10,000 times. Each permutation yields a count of supra-threshold voxels, which is used to create the null distribution.[11] Moreover, the permutation test did not shuffle the position of the voxels relative to each other, thus preserving the spatial autocorrelation across permutations. Such “extent-based inferences” in non-parametric permutation testing were interpreted simply as a rejection of the null hypothesis at a conventional alpha of 0.05 ($p_{corrected} <0.05$) or a failure to reject the null hypothesis ($p_{corrected} >0.05$). In other words, $p_{corrected} < 0.05$ implies that k (supra-threshold voxel count) from real data was in the top 5% of the null distribution from 10,000 k values from shuffled datasets.

Of note, the main effect of age was not statistically significant in the voxelwise analysis

© 2025 Perlman G et al. *JAMA Network Open*.

(5 positive-sign supra-threshold voxels, $p_{corrected}$ = 0.89, 262 negative-sign supra-threshold voxels, $p_{corrected}$ = 0.11), perhaps due to the narrow age range of the sample. Nonetheless, age was included as a covariate in each model for completeness given current conventions in the field[12]. To test association with the positive valence system (Extraversion), we repeated the voxelwise regression analysis for trait extraversion acquired at the time of the scan ("At MRI"), as well as for trait extraversion acquired at study baseline (many years before NM-MRI was acquired and prior to onset of depression; e.g., "At W1").

The MNI coordinates for specific sub nuclei within the SN-VTA complex were identified by probabilistic atlas at a threshold of 50%[13]: ventral tegmental area (VTA; 29 voxels), SN pars compacta (SNc; 249 voxels), SN pars reticulata (SNr; 567 voxels), and parabrachial pigmented nucleus (PBP; 128 voxels). The remaining voxels in the SN-VTA mask have lower probability of inclusion in these four regions and are therefore classified as Other SN (n=1319 voxels).

© 2025 Perlman G et al. *JAMA Network Open*.

eResults

Categorical Analysis: Association between NM-MRI Contrast and Chronic Depression Groups

ROI analysis: To complement the whole-mask voxelwise analyses described in the manuscript, we also examined the association between Chronic Depression Groups and NM-MRI contrast using the mean of voxels in the SN-VTA mask. Depression Groups differed on the mean contrast in whole mask ($F(2,101) = 3.11$, $p = 0.03$; See Figure 2b). By midbrain subnuclei mask, group differences were present in the SNc ($F(2,101) = 2.82$ $p = 0.04$), VTA ($F(2,101) = 3.49$, $p = 0.02$), and PBP ($F(2,101) = 3.17$, $p = 0.03$), but not SNr ($F(2,101) = 1.67$, $p = 0.18$) (eTable 2). Bonferroni-adjusted post-hoc tests (e.g., threshold $p < 0.0167$) showed that Chronic Depression was associated with less NM-MRI contrast (relative Non-Chronic Depression and Never Depressed Groups) for the Whole Mask, VTA, and PBP. Chronic Depression was associated with less NM-MRI contrast relative to Non-Chronic Depression for SNc.

Dimensional Analysis: Association between NM-MRI Contrast and log transformed Depression Months

ROI analysis: The negative association between mean NM-MRI contrast in whole mask and log 10 transformed Depression Months was significant ($B = -1.26$, $r = -0.38$, $p < 0.01$). Among subnuclei, log10 transformed Depression Months were significantly negatively associated with mean contrast in VTA ($B = -2.61$, $r = -0.59$, $p<0.01$), PBP ($B = -2.21$, $r = -0.48$, $p<0.01$), and SNc ($B = -1.30$, $r= -0.32$, $p < 0.01$), but not mean SNr ($B = -0.35$, $r = -0.10$, $p=0.55$). (See eTable 3).

Dimensional Analysis: Association between NM-MRI Contrast and untransformed Depression Months

Voxelwise Analysis: For thoroughness, we repeated analysis using the raw (not log10 transformed) Depression Months. The Depression Months was significantly negatively correlated with the NM-MRI contrast: longer depression chronicity was associated with lower contrast (0 positive supra-threshold voxels, $p_{corrected} = 0.99$; 1154 negative-sign supra-threshold voxels, $p_{corrected} <0.001$). The point-biserial LOOr (effect size) was robust, LOOr = -0.60.

ROI analysis: The negative association between mean NM-MRI contrast in whole mask and Depression Months was significant ($B = -0.05$, $r = -0.56$, $p < 0.001$). Among subnuclei, Depression Months were significantly negatively associated with mean contrast in VTA ($B = -0.08$, $r = -0.67$, $p<0.001$), PBP ($B = -0.07$, $r=0.55$, $p<0.001$), and SNc ($B = -0.06$, $r= -0.51$, $p=0.001$), but not mean SNr ($B = -0.03$, $r = -0.26$, $p=0.11$). (See eTable 3).

© 2025 Perlman G et al. *JAMA Network Open*.

Dimensional Analysis: Association between NM-MRI Contrast and Extraversion

ROI analysis: The positive association between mean NM-MRI contrast in whole mask and Extraversion was significant ($B = 0.13$, $r = 0.31$, $p < 0.01$). Among subnuclei, Depression Months were significantly negatively associated with mean contrast in VTA ($B = 0.21$, $r = 0.37$, $p<0.01$), PBP ($B = 0.15$, $r=0.30$, $p<0.01$), and SNc ($B = 0.01$, $r= 0.27$, $p=0.01$), but not mean SNr ($B = 0.06$, $r = 0.15$, $p=0.13$). (See eTable 4).

Test of Cumulative Substance Use (factor score) as Confound

We considered whether cumulative substance use accounted for the association between chronic depression and NM-MRI contrast**.** In brief, cumulative substance use was operationalized as the first common factor among five lifetime substance use variables: number of drug classes tried, number of alcohol intoxications in lifetime, number of days of cannabis use in lifetime, number of cigarettes per day during the heaviest month, and Any DSM-5 Substance Use Disorder, as described elsewhere[14]. The categorical depression groups differed on cumulative substance use (factor score), $F(2,102) = 4.20$, $p = 0.02$. Comparison of group means revealed that the non-chronic depression group reported the most substance use on average, followed by the Never Depressed group, and then the Chronic Depression group. Of note, the voxelwise association between Depression Group and NM-MRI contrast was not meaningfully different when adding cumulative substance use (factor score) as a covariate (617 supra-threshold voxels, $p_{corrected} = 0.02$). In addition, cumulative substance use (factor score) remained significant when controlling for Depression Group and Age (672 supra-threshold voxels, $p_{corrected} = 0.03$). eTable 5 presents the results of region-of-interest analysis with Depression Group, Cumulative Substance use, and Age entered as simultaneous predictors. In addition, the relationship with Extraversion was statistically significant when controlling for Age and Cumulative Substance Use (factor score) (886 supra-threshold voxels, $p_{corrected} <0.01$). Thus, it does not appear if cumulative substance use history accounts for the negative association between chronic depression and NM-MRI contrast or the positive association between extraversion and NM-MRI contrast.

Test of Motion/Data Quality as Confound

We conducted two additional tests to examine the possibility of motion confounding our results.

First, the number of “approved” volumes (e.g. non-artifact volumes used in subject average) did not differ by Depression Group ($F(2,102) = 0.33$, $p = 0.72$): Never Depressed ($M = 10.31$, $SD = 1.62$), Non-Chronic (M=

© 2025 Perlman G et al. *JAMA Network Open*.

10.07, SD = 2.02), and Chronic (M = 10.22, SD = 1.30). The number of “approved” volumes was also weakly and non-significantly associated with Depression Months (r = -0.05, p = 0.78, n = 37) and Extraversion (r = -0.01, p = 0.96, n = 105). Second, we computed the mean total framewise displacement (FD) per “approved” volume for each subject. Mean FD did not differ by Depression Group (F(2,102) = 0.19, p = 0.83): Never Depressed (Mean = 1.63, SD 1.03), Non-Chronic (M = 1.63, SD = 0.82), and Chronic (M = 1.89, SD = 0.58). FD was also weakly associated with Depression Months (r = 0.21, p = 0.21, n = 37) and Extraversion (r = -0.06, p = 0.51, n = 105). Based on this evidence, we are unable to corroborate the hypothesis that differences in motion between Depression Groups explains observed differences in NM-MRI contrast.

eReferences

1. Keller MB, Lavori PW, Friedman B, Nielsen E, Endicott J, McDonald-Scott P, Andreasen NC. The Longitudinal Interval Follow-up Evaluation. A comprehensive method for assessing outcome in prospective longitudinal studies. *Arch Gen Psychiatry.* 1987;44(6):540-548.
2. Klein DN. Diagnosis and classification of depressive disorders. In: Olino JWPTM, ed. *APA Handbook of Depression*. Washington, DC: American Psychological Association Press; in press.
3. Klein DN, Perlman G, Feltman SM, Kotov R. Preonset predictors of chronic-intermittent depression from early adolescence to early adulthood. *J Psychopathol Clin Sci.* 2023;132(6):694-703.
4. Mondimore FM, Zandi PP, MacKinnon DF, McInnis MG, Miller EB, Schweizer B, Crowe RP, Scheftner WA, Weissman MM, Levinson DF, DePaulo JR, Jr., Potash JB. A comparison of the familiality of chronic depression in recurrent early-onset depression pedigrees using different definitions of chronicity. *J Affect Disord.* 2007;100(1-3):171-177.
5. Silver J, Olino TM, Carlson GA, Klein DN. Offspring of Mothers With Histories of Chronic and Non-chronic Depression: Symptom Trajectories From Ages 6 to 15. *Front Psychiatry.* 2020;11:601779.
6. Rammstedt B, John OP. Measuring personality in one minute or less: A 10-item short version of the Big Five Inventory in English and German. *Journal of Research in Personality.* 2007;41(1):203-212.
7. John OP, Naumann, L. P., & Soto, C. J. Paradigm Shift to the Integrative Big-Five Trait Taxonomy: History, Measurement, and Conceptual Issues. In: O. P. John RWR, & L. A. Pervin, ed. *Handbook of personality: Theory and research*. New York, NY: Guilford Press.; 2008:114-158.
8. Salzman G, Kim J, Horga G, Wengler K. Standardized Data Acquisition for Neuromelanin-sensitive Magnetic Resonance Imaging of the Substantia Nigra. *J Vis Exp.* 2021(175).
9. Wengler K, He X, Abi-Dargham A, Horga G. Reproducibility assessment of neuromelanin-sensitive magnetic resonance imaging protocols for region-of-interest and voxelwise analyses. *Neuroimage.* 2020;208:116457.
10. Cassidy CM, Zucca FA, Girgis RR, Baker SC, Weinstein JJ, Sharp ME, Bellei C, Valmadre A, Vanegas N, Kegeles LS, Brucato G, Kang UJ, Sulzer D, Zecca L, Abi-Dargham A, Horga G. Neuromelanin-sensitive MRI as a noninvasive proxy measure of dopamine function in the human brain. *Proc Natl Acad Sci U S A.* 2019;116(11):5108-5117.
11. Eklund A, Nichols TE, Knutsson H. Cluster failure: Why fMRI inferences for spatial extent have inflated false-positive rates. *Proc Natl Acad Sci U S A.* 2016;113(28):7900-7905.
12. Wengler K, Trujillo P, Cassidy CM, Horga G. Neuromelanin-sensitive MRI for mechanistic research and biomarker development in psychiatry. *Neuropsychopharmacology.* 2024;50(1):137-152.
13. Pauli WM, Nili AN, Tyszka JM. A high-resolution probabilistic in vivo atlas of human subcortical brain nuclei. *Sci Data.* 2018;5:180063.
14. Perlman G, Wengler K, Moeller SJ, Kotov R, Klein DN, Weinstein JJ, Horga G, Abi-Dargham A. Association of Neuromelanin-Sensitive MRI Signal With Lifetime Substance Use in Young Women. *Am J Psychiatry.* 2024;181(11):997-1005.

© 2025 Perlman G et al. *JAMA Network Open*.

eTable 1. Sample Bias Analysis

	MRI sample			Not Included in MRI sample			Statistical test
	n	M	SD	n	M	SD	
Extraversion at W1	118	22.53	4.48	432	22.59	5.27	t(165.99)=0.10, p = 0.91
Extraversion at W6[%]	117	21.36	5.55	342	21.51	5.25	t(192.01)=0.25, p = 0.80
Age	118	14.32	0.63	432	14.39	0.62	t(184) = 1.01, p=0.32
Household Income at W1	118	6.57	1.73	430	6.04	2.16	t(227.2) = -2.79, p = <0.01
	n	%		n	%		
Non-Hispanic	106	89.8		384	88.9		$X^2(2)$ = 0.01, p = 0.90
Caucasian	111	94.1		371	85.9		$X^2(2)$ = 5.00, p = 0.03
Depression Group							$X^2(2)$ = 0.48, p = 0.79
Never Depressed	76	64.4		260	60.2		
Non-Chronic Depression	32	27.1		129	29.9		
Chronic Depression	10	8.5		36	8.3		

Note: Household Income was rated by the participating parent at baseline on a 9 point ordinal scale where 1 = “Less than $20k”, 2 = $20k-$40k”, 3 = $40k-$60k”, 4 = $60k-$80k”, 5 = $80k-$100k”, 6 = $100k-$120k”, 7 = $120k-$150k”, 8 = $150k-$180k”, 9 = More than $180k; % = “Extraversion at Wave 6” is the most proximal assessment of extraversion to the time of MRI.

© 2025 Perlman G et al. *JAMA Network Open*.

eTable 2. Region-of-Interest Analysis for Depression Groups

	Never		Non-Chronic		Chronic			
	M	SD	M	SD	M	SD	Statistical Test	Group Comparisons
Whole Mask	10.68	2.43	10.90	1.65	8.69	1.52	F(2,101) = 3.11, p = 0.03	3 < 1, 3 < 2
SNc	14.28	2.79	14.83	2.10	12.27	1.67	F(2,101) = 2.82, p = 0.04	3 < 1
SNr	9.66	2.47	9.83	2.04	8.64	1.53	F(2,101) = 1.67, p = 0.18	3 < 1, 3< 2
VTA	15.63	3.25	16.21	2.05	12.76	1.96	F(2,101) = 3.49, p = 0.02	3 < 1, 3< 2
PBP	13.61	2.76	14.13	2.38	11.18	1.88	F(2,101) = 3.17, p = 0.03	3 < 1, 3< 2

Note: All analyses controlling for age. Group Comparisons listed were statistically significant given Bonferroni correction (p < 0.0167). Group 1 = Never Depressed, Group 2 = Non-Chronic Depression, Group 3 = Chronic Depression.

© 2025 Perlman G et al. *JAMA Network Open*.

eTable 3. Region-of-Interest Analysis for Depression Months

	Untransformed				Log10 Transformed			
	estimate	p	r	95% CI	estimate	p	r	95% CI
Whole Mask	-0.05	<0.01	-0.56	-0.75, -0.30	-1.26	0.02	-0.38	-0.63, -0.06
SNc	-0.06	<0.01	-0.51	-0.71, -0.22	-1.30	0.05	-0.32	-0.58, 0.00
SNr	-0.03	0.10	-0.27	-0.55, 0.05	-0.35	0.55	-0.10	-0.41, 0.23
VTA	-0.08	<0.01	-0.67	-0.82, -0.44	-2.61	<0.01	-0.59	-0.76, -0.32
PBP	-0.07	<0.01	-0.55	-0.74, -0.28	-2.21	<0.01	-0.48	-0.69, -0.18

Note: All analyses controlling for age

© 2025 Perlman G et al. *JAMA Network Open*.

eTable 4. Region-of-Interest Analysis for Extraversion

	estimate	p	r	95% CI
Whole Mask	0.13	<0.01	0.31	0.12,0.47
SNc	0.13	0.01	0.27	0.09,0.44
SNr	0.06	0.13	0.15	-0.04,0.33
VTA	0.21	<0.01	0.37	0.19,0.53
PBP	0.15	<0.01	0.30	0.11,0.46

Note: All analyses controlling for age

© 2025 Perlman G et al. *JAMA Network Open*.

eTable 5. Group Differences in Substance Use

		Statistical Test
Whole Mask		
	Depression Group	F(1,100) = 3.40, p = 0.04
	Cumulative Substance Use (factor score)	F(1,100) = 4.95, p = 0.03
SNc		
	Depression Group	F(1,100) = 2.68, p = 0.07
	Cumulative Substance Use (factor score)	F(1,100) = 4.42, p = 0.04
SNr		
	Depression Group	F(1,100) = 0.87, p = 0.42
	Cumulative Substance Use (factor score)	F(1,100) = 1.98, p = 0.16
VTA		
	Depression Group	F(1,100) = 4.09, p = 0.04
	Cumulative Substance Use (factor score)	F(1,100) = 6.68, p = 0.01
PBP		
	Depression Group	F(1,100) = 3.63, p = 0.03
	Cumulative Substance Use (factor score)	F(1,100) = 5.16, p = 0.03

Note: All analyses controlling for Age

© 2025 Perlman G et al. *JAMA Network Open*.

eFigure 1. Mosaic Displays of Grand Average Neuromelanin-Sensitive Magnetic Resonance Imaging (NM-MRI) Contrast From Axial, Coronal, and Sagittal Views

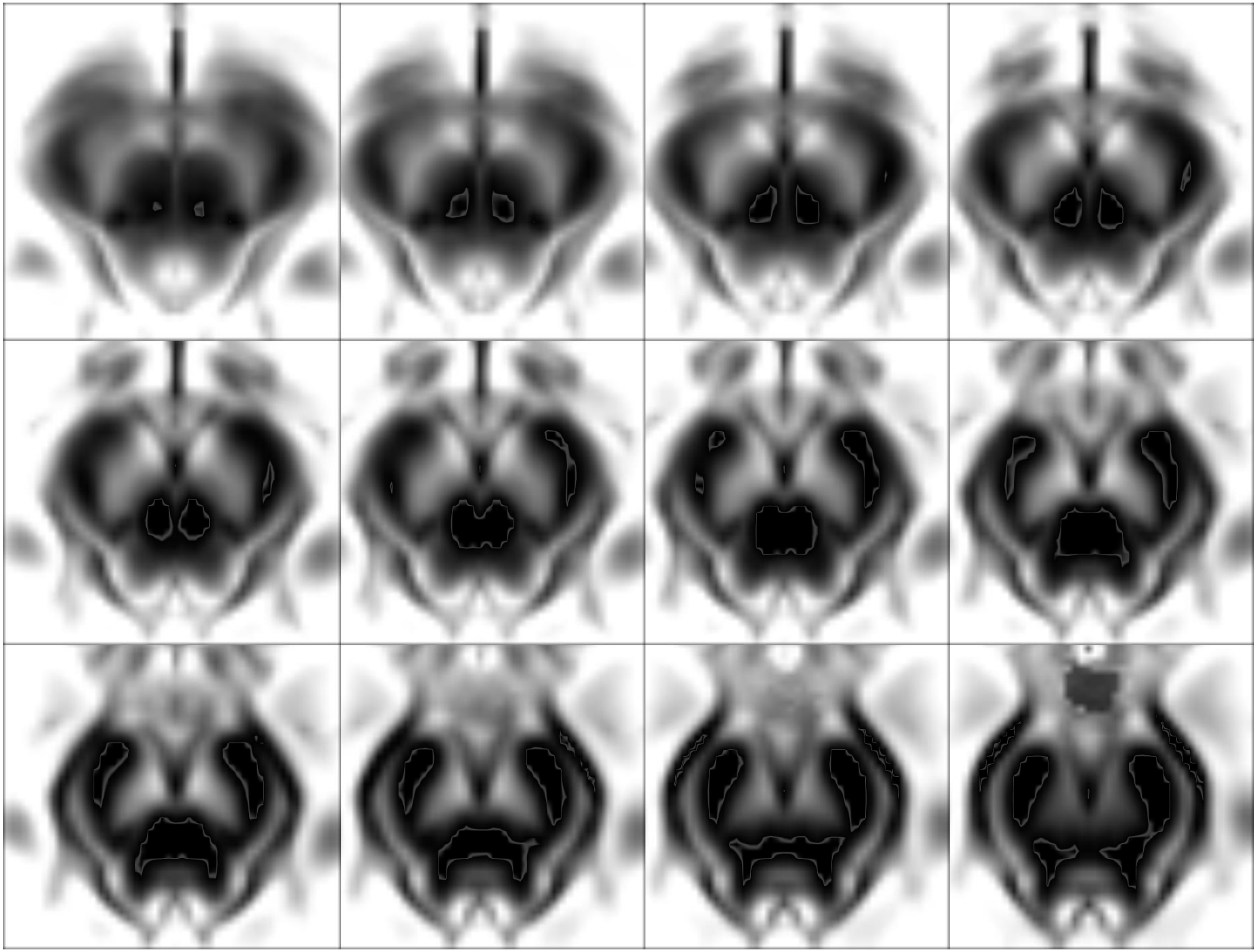

© 2025 Perlman G et al. *JAMA Network Open*.

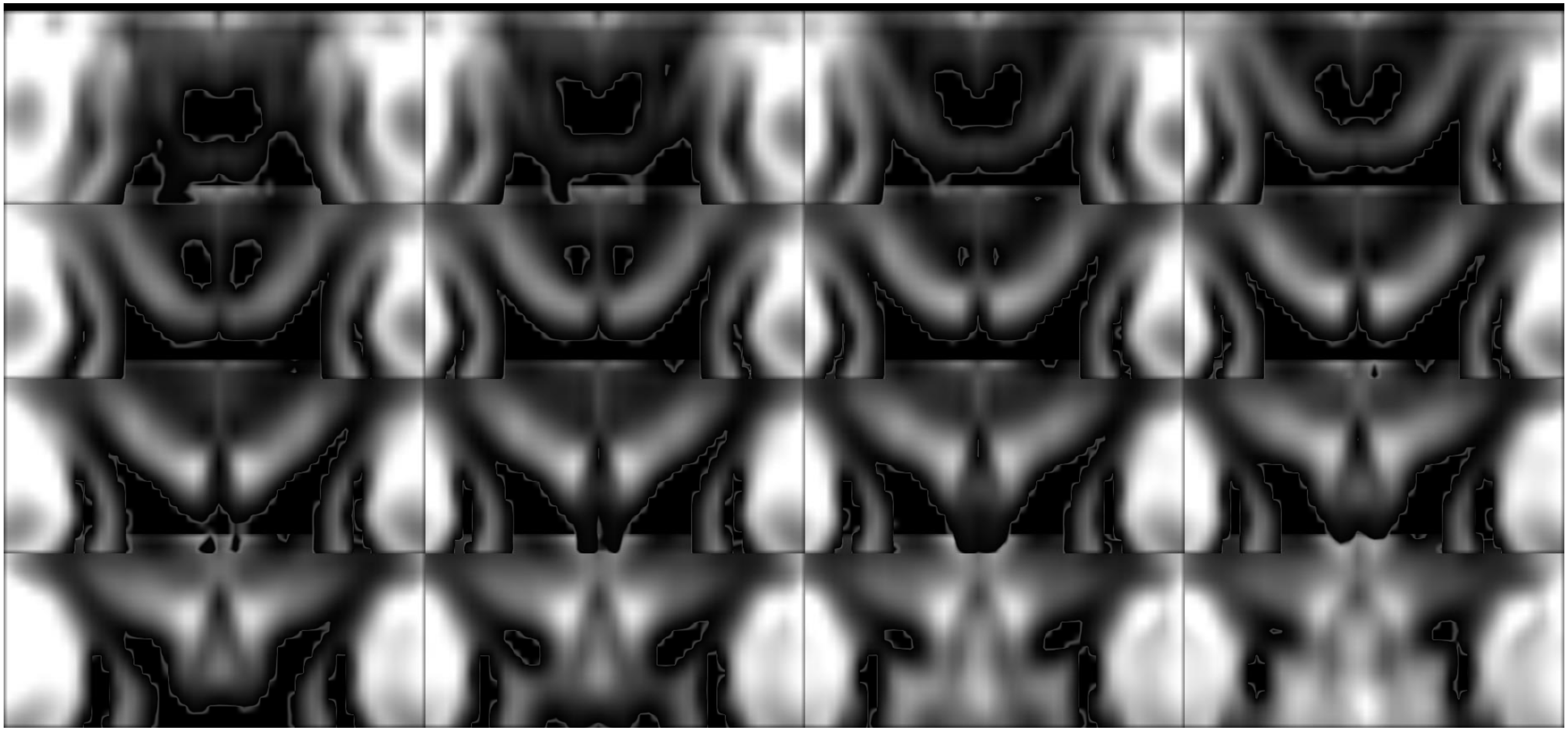

© 2025 Perlman G et al. *JAMA Network Open*.

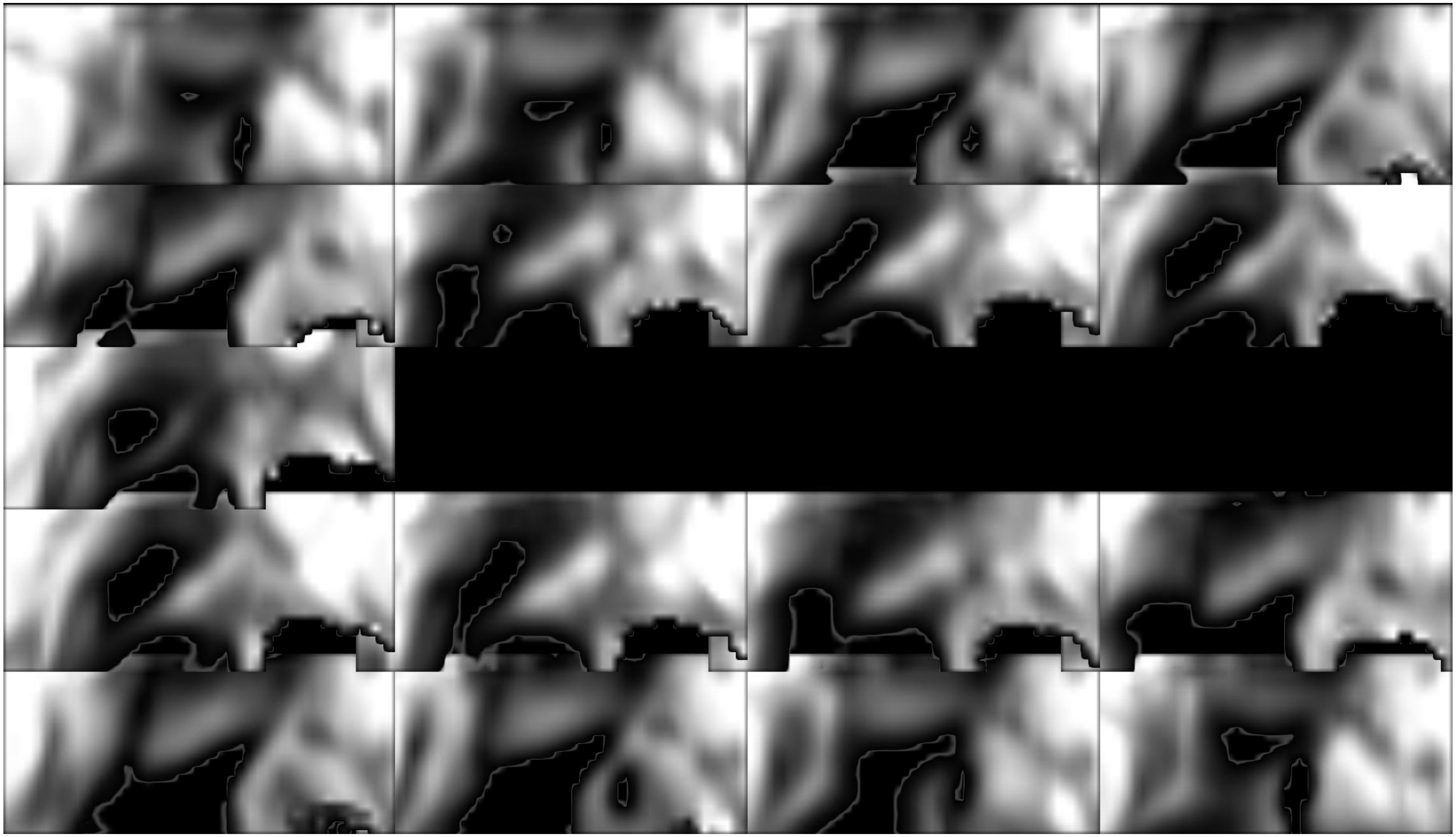

© 2025 Perlman G et al. *JAMA Network Open*.

eFigure 2. Mosaic Displays of Neuromelanin-Sensitive Magnetic Resonance Imaging (NM-MRI) Contrast From Axial, Coronal, and Sagittal Views With a *t*-Statistic Map From Voxelwise Analysis

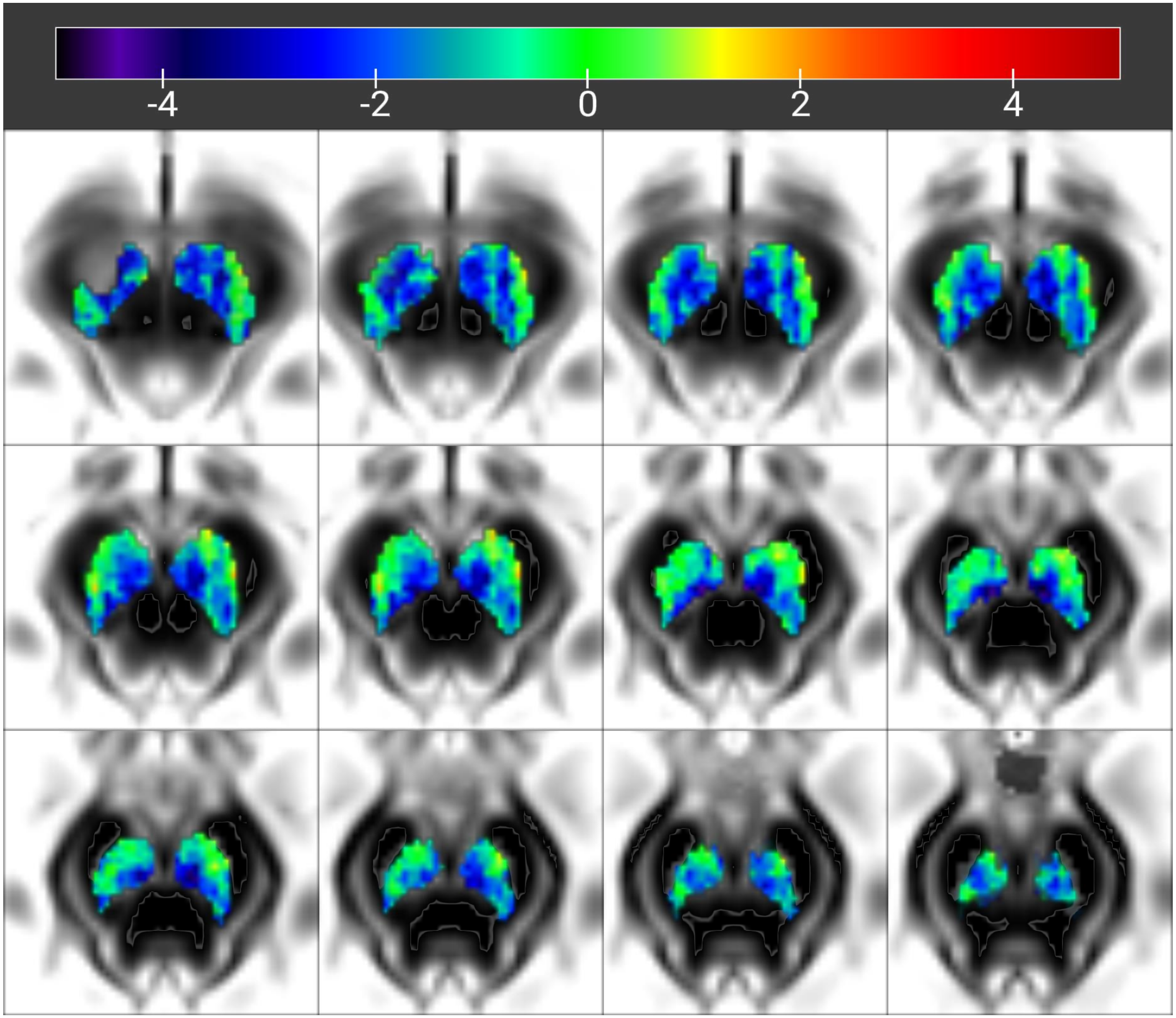

© 2025 Perlman G et al. *JAMA Network Open*.

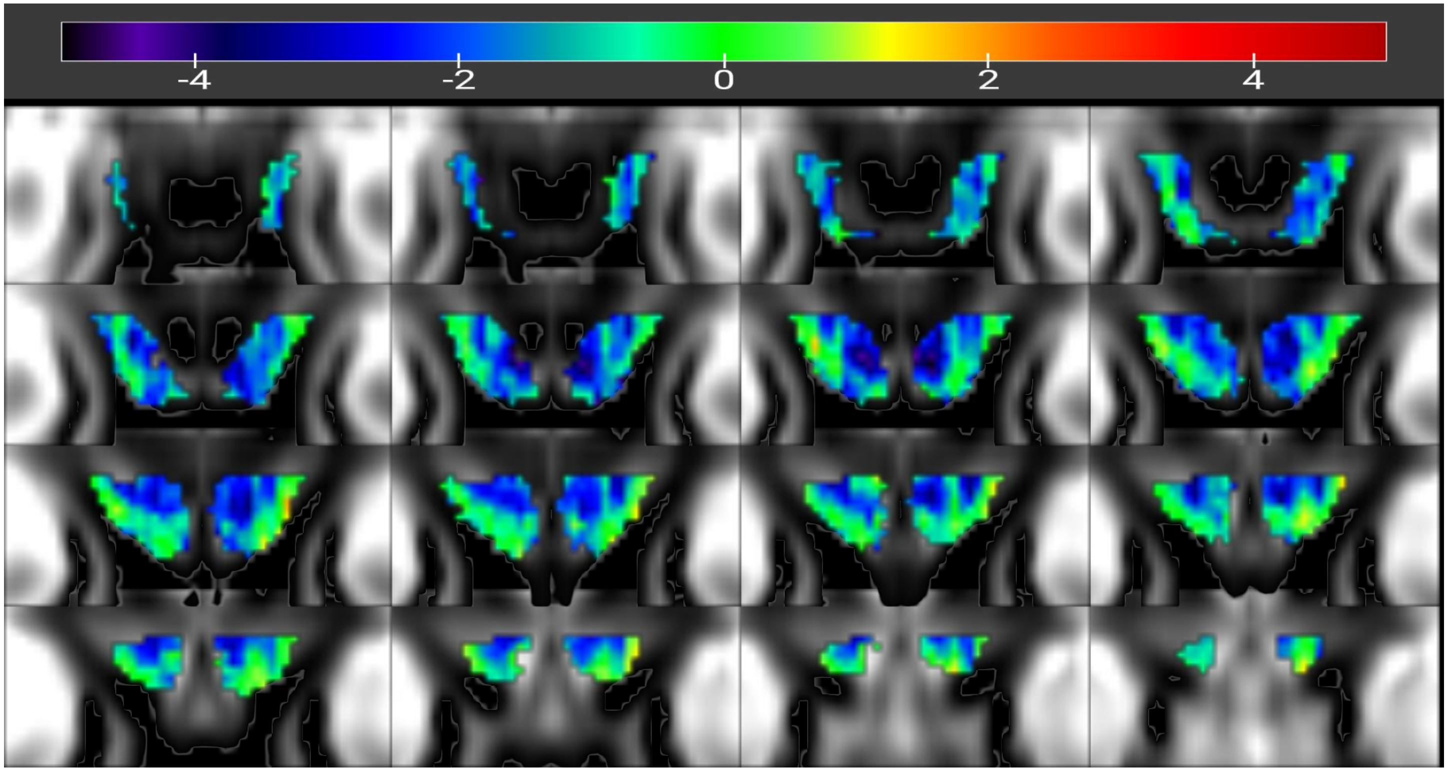

© 2025 Perlman G et al. *JAMA Network Open*.

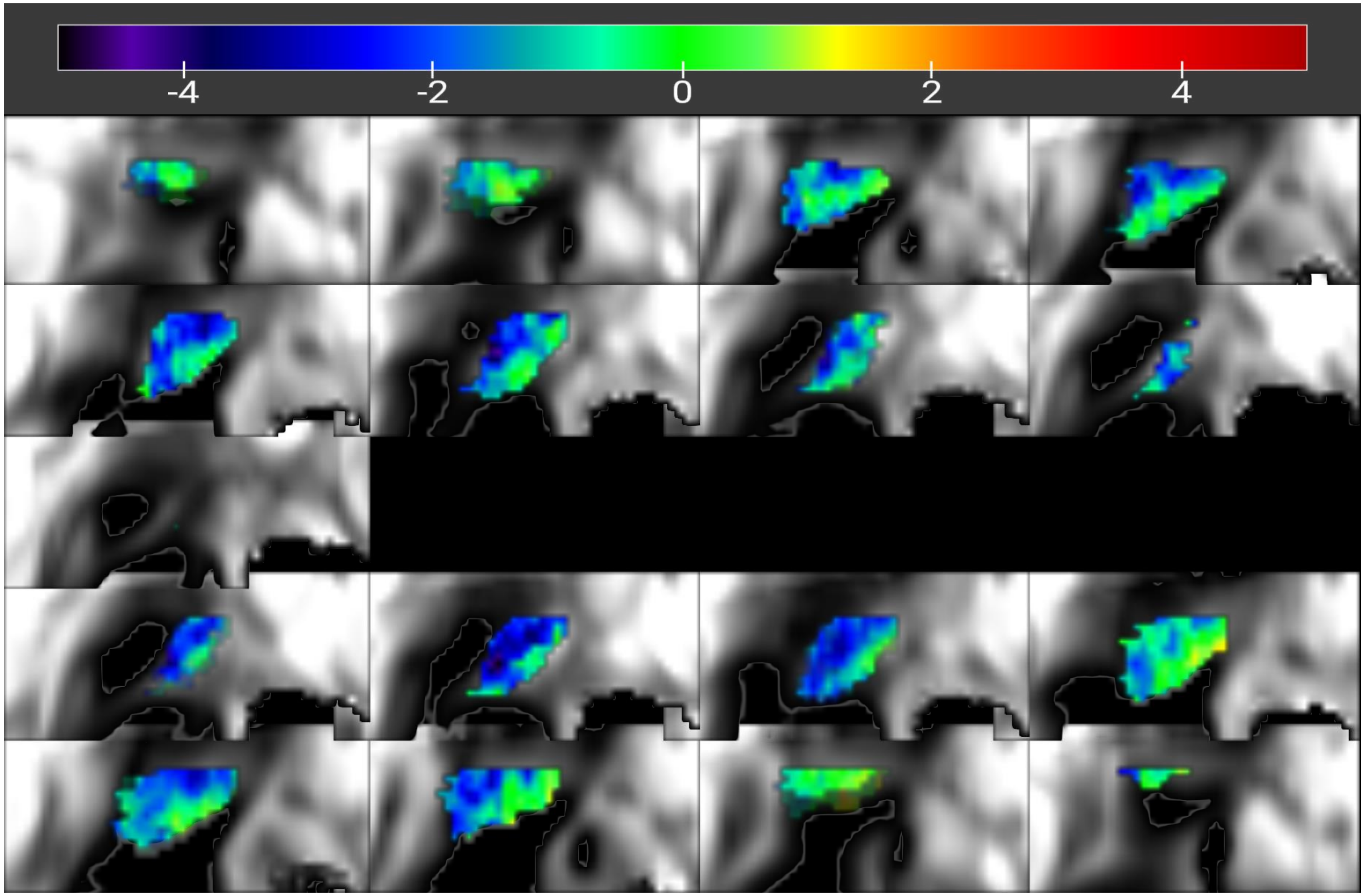

© 2025 Perlman G et al. *JAMA Network Open*.

eFigure 3. Histogram of Significant Voxels From Association Between Neuromelanin-Sensitive Magnetic Resonance Imaging (NM-MRI) Contrast and Depression Months

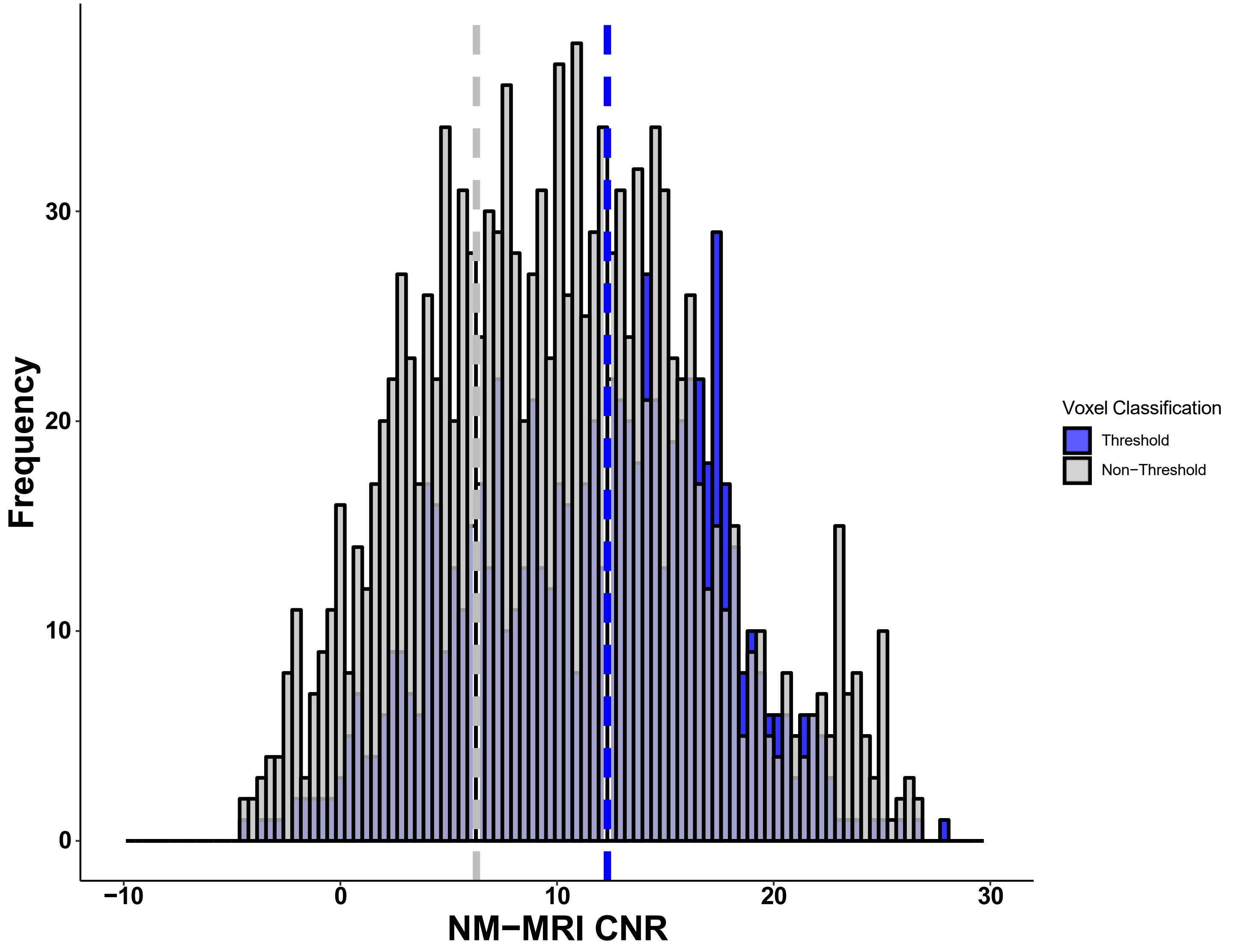

© 2025 Perlman G et al. *JAMA Network Open*.
