# Supplementary material for: Neuromelanin-Sensitive MRI Contrast and Chronic Depression in Young Women
Source: JAMA Netw Open. 2025 Sep 23;8(9):e2533339. doi: 10.1001/jamanetworkopen.2025.33339 (PMC12457983; doi:10.1001/jamanetworkopen.2025.33339)
Supplement: Supplement 2. — Data Sharing Statement [file jamanetwopen-e2533339-s002.pdf]

Data Sharing Statement

Perlman. Neuromelanin-Sensitive MRI Contrast and Chronic Depression in Young Women. *JAMA Netw Open*. Published September 23, 2025. doi:10.1001/jamanetworkopen.2025.33339

Data
Data available: Yes
Data types: Deidentified participant data
How to access data: Data will be made available upon request in accordance with the guidelines set forth by the Stony Brook University Institutional Review Board.
When available: With publication

Supporting Documents
Document types: Statistical/analytic code
How to access documents: Scripts will be made available upon request.
When available: With publication

Additional Information
Who can access the data: Data will be made available upon request in accordance with the guidelines set forth by the Stony Brook University Institutional Review Board.
Types of analyses: Data will be made available upon request in accordance with the guidelines set forth by the Stony Brook University Institutional Review Board.
Mechanisms of data availability: with a signed data access agreement
Any additional restrictions: n/a
